# Anticorrosion, Thermal Degradation, and Hydrophobic Performances of Graphene/${\mathrm{TiO}}_{2}$ Nanocomposite Coatings

**DOI:** 10.3390/polym15112428

**Published:** 2023-05-23

**Authors:** Sachin Sharma Ashok Kumar, Nujud Badawi Mohammed, Osamah Alduhaish, Kasi Ramesh, Subramaniam Ramesh, Mujeeb Khan, Baji Shaik, Syed. F. Adil

**Affiliations:** 1 Centre for Ionics University of Malaya, Department of Physics, Faculty of Science, Universiti Malaya, Kuala Lumpur 50603, Malaysia; 2 Chemistry Department, College of Science, King Saud University, Riyadh 11451, Saudi Arabia; 3 School of Chemical Engineering, Yeungnam University, Gyeongsan 38541, Republic of Korea

**Keywords:** graphene nanofillers, TiO_2_ nanoparticles, acrylic-epoxy polymer matrix, electrochemical impedance spectroscopy (EIS), thermogravimetric analysis (TGA), field emission scanning electron microscopy (FESEM)

## Abstract

Globally, researchers have devoted consistent efforts to producing excellent coating properties since coating plays an essential role in enhancing electrochemical performance and surface quality. In this study, TiO_2_ nanoparticles in varying concentrations of 0.5, 1, 2, and 3 wt.% were added into the acrylic-epoxy polymeric matrix with 90:10 wt.% (90A:10E) ratio incorporated with 1 wt.% graphene, to fabricate graphene/${\mathrm{TiO}}_{2}$ -based nanocomposite coating systems. Furthermore, the properties of the graphene/${\mathrm{TiO}}_{2}$ composites were investigated by Fourier-transform infrared spectroscopy (FTIR), thermogravimetric analysis (TGA), ultraviolet-visible (UV-Vis) spectroscopy, water contact angle (WCA) measurements, and cross-hatch test (CHT), respectively. Moreover, the field emission scanning electron microscope (FESEM) and the electrochemical impedance spectroscopy (EIS) tests were conducted to investigate the dispersibility and anticorrosion mechanism of the coatings. The EIS was observed by determining the breakpoint frequencies over a period of 90 days. The results revealed that the ${\mathrm{TiO}}_{2}$ nanoparticles were successfully decorated on the graphene surface by chemical bonds, which resulted in the graphene/${\mathrm{TiO}}_{2}$ nanocomposite coatings exhibiting better dispersibility within the polymeric matrix. The WCA of the graphene/${\mathrm{TiO}}_{2}$ coating increased along with the ratio of ${\mathrm{TiO}}_{2}$ to graphene, achieving the highest CA of 120.85° for 3 wt.% of TiO_2_. Excellent dispersion and uniform distribution of the ${\mathrm{TiO}}_{2}$ nanoparticles within the polymer matrix were shown up to 2 wt.% of TiO_2_ inclusion. Among the coating systems, throughout the immersion time, the graphene/${\mathrm{TiO}}_{2}$ (1:1) coating system exhibited the best dispersibility and high impedance modulus values (${\left|Z\right|}_{0.01\;\mathrm{Hz}}$), exceeding $10^{10}$ Ω$\;{\mathrm{cm}}^{2}$.

## 1. Introduction

Corrosion is defined as the process of loss and destruction due to the chemical or electrochemical interaction between the metal substances and corrosive environments, which have resulted in the disintegration and deterioration of metal or their alloy materials [1,2,3]. Therefore, it is of great significance to exploit several suitable and effective anticorrosion strategies [4,5]. Today, coating applications have resulted in the enhancement of the wettability, adhesion, corrosion resistance, and surface properties of steel or other types of substrates in general. Furthermore, in relation to the economic benefits and growing environmental concerns, the coating industry has been driven to seek new technologies and materials to enhance the efficiency of the coatings. In addition, the corrosive environment, coating quality, properties of the coating/substrate interface, and the attributes of the substrate are some of the factors that affect the effectiveness of a coating [6]. Hence, polymer nanocomposites consisting of superior properties have been frequently employed and investigated for industrial applications (aircraft, marine, oil and gas, construction, etc.).

In order to control the corrosion-promotion activity of graphene, it is essential to obtain an insight into the mechanism of the corrosion-promotion occurrence [7]. Previously, Schriver and Zhou et al. reported that the observed corrosion-promotion process introduced by graphene played a significant role in the galvanic corrosion of graphene–metal couple [8,9]. In addition, by removing the necessary conditions for the phenomenon of the galvanic corrosion of the graphene–metal couple, for instance, aggressive electrolytes, potential difference, and electric connections between the graphene and metal, respectively, hence, this would result in achieving the inhibition of the corrosion-promotion activity of the graphene. Furthermore, the intrinsic properties of graphene and the metal substrate were known to be the fundamental cause of the graphene–metal galvanic corrosion, particularly for graphene-based polymer nanocomposite coatings [7]. In general, the thermodynamic stability and high conductivity (intrinsic properties of graphene) were the two crucial factors specifically for graphene possessing corrosion-promotion activity [8,9]. On the other hand, certain extrinsic factors resulting from the coating fabrication process could provide further assistance to the graphene in order to inhibit the corrosion-promotion activity: for instance, the coating defects and the connections between the graphene and metal, respectively. Despite the fact that the graphene-based polymer nanocomposite coatings enhanced the barrier properties and have exhibited high corrosion protection performances, however, it is also noteworthy to mention that the inclusion of graphene could possibly increase the bulk conductivity (some orders of magnitude) of an otherwise insulating polymer, thus resulting in an unacceptable corrosion-promotion activity of the polymer [7]. For instance, it was previously reported that the electrical percolation threshold of some polymers, namely poly (ethylene-2,6-naphthalate), thermoplastic polyurethane, polycarbonate, etc., were observed to be possibly lower compared to the critical graphene loadings [10,11,12,13,14]. In other words, graphene-based polymer coatings possess both superior barrier properties and high conductivity, but when the coatings suffer from defects, they are susceptible to accelerating the rate of metal corrosion. In short, the removal of the factors mentioned above via the emergence of new approaches is the key to inhibiting the corrosion-promotion activity of graphene-based coatings.

Furthermore, organic coatings can be easily fabricated on metal substrates, thus playing an essential role in inhibiting the corrosion process [15]. For instance, endowing to the high bond energy and hydrophobic characteristics, polydimethylsiloxane (PDMS) with Si-O-Si bond and −${\mathrm{CH}}_{3}$ as main chain and side chains have exhibited excellent hydrophobicity, heat resistance, and flexibility [1,16]. In addition, PDMS has been frequently utilized in a wide range of applications, namely, anticorrosive coatings, flexible devices, cell sheet engineering, humidity sensors, etc. On the other hand, in long-term usage, pure PDMS coatings easily encounter electrolysis and degradation due to the penetration of electrolytes and water via the existing microcracks and micropores from its structure [1,17]. In other words, the incorporation of nanoparticles into the PDMS composite coating is favorable to comprehensively enhance the anticorrosion performance of the organic coatings [17].

In the year 2004, a new generation material, graphene, was first isolated by simple mechanical exfoliation [18]. Graphene is two-dimensional (2D) that consists of a single-layer crystal honeycomb lattice structure that is made up of the tightly-packed ${\mathrm{sp}}^{2}$ bonded carbon atoms [19,20,21,22,23]. Furthermore, graphene has been known to be the main building block of other important carbon allotropes and due to its unique structure, these carbon atoms form an excellent electrons carrier space, thus exhibiting a high electron mobility (250,000 ${\mathrm{cm}}^{2}$/V), ballistic transport and quantum hall effect at room temperature, respectively [15]. Besides, it was previously reported that graphene has high mechanical properties, for instance, exhibiting Young’s modulus and tensile strength of 1 TPa and 130 GPa, and the mechanical properties vary according to the number of graphene layers and the internal defects of the graphene layers, respectively [15]. Moreover, at room temperature, the highest thermal conductivity exhibited by the graphene was approximately 5000 W/mK [15]. Furthermore, due to its superior properties, graphene was reported to be a potential and efficient nanofiller to remarkably improve the performance of the high-quality polymer matrix nanocomposite coating. In addition, graphene has been identified as a high water and oil-repellent material. Therefore, the high water and oil resistance property makes graphene an ideal material for coating applications [19]. To support this finding, Lin et al. investigated the wear resistance and the frictional properties of multi-layered graphene films using atomic force microscopy (AFM) [24]. Here, it was reported that these graphene films exhibited exceptional wear resistance and frictional properties. In other words, the graphene has a high tendency to completely cover the coated substrate and protect the coating against scratches or other physical damage on the applied substrate. Interestingly, due to being chemically inert, graphene was also proven to be an effective corrosion barrier material, thus, enhancing the anti-corrosion property of a coating system [1,15]. For instance, Sun and Cui et al. investigated the modified graphene nanosheets with molecular-sized PDMS-based epoxy coating [25]. Here, it was reported that the graphene nanosheets homogenously dispersed in common paint solvents and further demonstrated the ability to reinforce the anticorrosion performance of the coatings.

At a very low weight percentage, the graphene and GO nanoparticles can be well dispersed and exfoliated in the polymeric matrix due to their high surface area and strong Van der Waals interactions [26]. However, at a relatively higher loading content, the nanoparticles will have the tendency to form severe aggregations, thus restricting its predicted properties within the polymer matrix from being achieved [27,28,29,30]. Hence, it is essential to address these issues and to improve the dispersion and exfoliation of the sheet filler. Lately, this goal has been successfully achieved by incorporating a variety of processing techniques such as organic modification, surface grafting, decorating, and in situ polymerization of nanoparticles into the polymeric matrix [26]. Moreover, as resin fillers, the nanoparticles have been wide employed to block the micropores and enhance the corrosion resistance and mechanical properties of the resins due to its small size effect and surface effect, respectively [31,32]. Globally, researchers have reported that the inclusion of inorganic nanoparticles on the surface of graphene and GO have resulted in the dispersion of the graphene and GO sheets effectively increasing, thus, reducing the aggregation effect [33,34,35,36]. In addition, for surface decoration, silica, titanium dioxide (${\mathrm{TiO}}_{2}$), zinc oxide (ZnO), silicon dioxide (${\mathrm{SiO}}_{2}$), and aluminum oxide (${\mathrm{Al}}_{2}O_{3}$) are some of the inorganic nanoparticles that are frequently used to develop high anticorrosion performance nanocomposite coatings [37]. On the other hand, Martins and Zhang et al. reported that the inclusion of ${\mathrm{TiO}}_{2}$ nanoparticles into graphene and GO-based nanocomposite coatings have resulted in the enhancement of optical and electrical properties specifically for photocatalysis [38,39]. For instance, the G/${\mathrm{TiO}}_{2}$ and GO/${\mathrm{TiO}}_{2}$ nanocomposites that have been fabricated via hydrothermal, solvothermal, and one-step colloidal blending approaches have exhibited high photocatalytic efficiency. This was due to the increased adsorption of pollutants onto the nanoparticles surface and the increased separation of charge carriers which effectively inhibited the recombination, respectively [38]. Moreover, these findings were commonly reported in other literatures whereby these nanocomposites resulted the band gap to reduce and simultaneously promoting separation of charge carriers at the interfaces [40,41,42]. Besides, Ahmad et al. stated that the photocatalytic degradation method effectively enabled the nanocatalyst to oxidize organic compounds into inorganic matters (e.g., oxygen and water) without any intermediate pollutants [43]. Furthermore, it was also reported that ${\mathrm{TiO}}_{2}$ along with other types of metal oxide (ZnO, NiO, CuO, etc.) nanoparticles acted as photocatalysts and played a vital role in the removal of methylene blue from polluted water [43]. Moreover, graphene and its nanomaterials-based metal oxides nanocomposites have also shown its significance and versatility as heterogenous catalyst for other applications including glucose sensor and antibacterial activity as well as the catalytic reduction of nitrogen oxides [44,45]. Hence, it is of great interest to explore the dispersion properties of the inclusion of different loading rates of ${\mathrm{TiO}}_{2}$ nanoparticles into the graphene-based acrylic-epoxy polymer nanocomposite coatings, which resulted in the comprehensive enhancement of surface wettability and the anticorrosion performance of the coatings [46].

Therefore, in this communication, the organic-inorganic based nanocomposite coating systems consisting of the acrylic-epoxy polymeric matrix, PDMS, 1 wt.% of graphene along with different loading rates of ${\mathrm{TiO}}_{2}$ nanoparticles (0.5–3 wt.%) were fabricated via the solution intercalation method with high hydrophobicity and superior electrochemical properties. In terms of characterization, FTIR was used to determine the chemical structure and the cross-linking occurrence of the coating films. The TGA was used to evaluate the thermal stability of the developed coatings. The UV-Vis spectroscopy and WCA instrument were utilized to determine the optical transmittance and the surface wettability of the coatings. The adhesion quality of the nanocomposite coatings to the steel substrate was evaluated by the CHT method. The influence of embedding ${\mathrm{TiO}}_{2}$ nanoparticles within the graphene-based polymeric matrix on the anticorrosion performance of the resulting coatings were investigated using the EIS tests. Finally, the FESEM was employed to closely investigate the dispersion of ${\mathrm{TiO}}_{2}$ nanoparticles within the polymeric matrix. Overall, due to the graphene sheet structure and its extraordinary mechanical properties, the results of the experiment revealed that graphene/${\mathrm{TiO}}_{2}$ nanocomposite coatings can effectively enhance the corrosion resistance and wear resistance of the coating. Hence, this study is of great significance for the practical application of graphene decorated with ${\mathrm{TiO}}_{2}$ nanoparticles, particularly in the anticorrosion field.

## 2. Materials and Methods

### 2.1. Materials

Without any further purification, all the listed chemicals were used as received from the suppliers to fabricate the hybrid polymer nanocomposite coatings. Acrylic polyol resin, denoted as A and its curing agent aliphatic poly-isocyanate resin (NCO) were purchased from Synthese, Selangor, Malaysia and Bayer Material Science, Germany, respectively. Epoxy resin, denoted as E, produced from bisphenol A and epichlorohydrin, was purchased from ASA Chemicals, Malaysia. Tables S1–S3 illustrate the material specifications and other chemical properties of the acrylic resin and its curing agent (NCO) and epoxy resin, respectively. Hydroxyl-terminated polydimethylsiloxane (HT-PDMS) with a viscosity of 750 cSt and a density of 0.97 g/mL at 25 °C was obtained from Sigma-Aldrich, Selangor, Malaysia and was used as a modifier. Isophorone diamine (IPDA) was used to create cross-linking during the coating blending process. The hydrophobic graphene nanoplatelets in powder form (PCode: 806668-25G) composed of carbon (>95 wt.%) and oxygen (<2 wt.%) and bulk density of 0.04 g/mL were purchased from Sigma-Aldrich, Malaysia. Titanium dioxide (${\mathrm{TiO}}_{2}$) anatase nanopowder, with a particle size of less than 25 nm and density of 3.9 g/mL at 25 °C, was purchased from Sigma-Aldrich, Malaysia and were used as reinforcing inhibitors.

### 2.2. Preparation of the Nanocomposite Coatings

**Figure 1:**
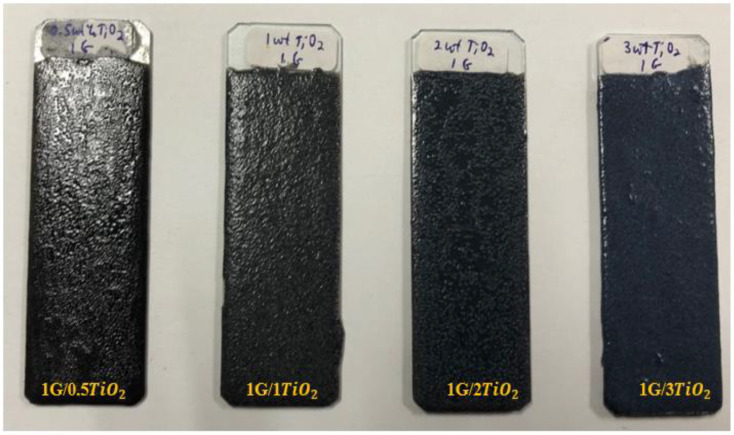
The coated glass substrates for each corresponding coating system, respectively.

This preparation was conducted to further investigate the effects of graphene nanofillers along with the inclusion of ${\mathrm{TiO}}_{2}$ nanoparticles as inhibitors into the polymer coatings. The ratio of acrylic-epoxy resins (90:10 wt.%) was measured accordingly using a weight scale and transferred into a beaker. Furthermore, four different nanocomposite coating samples consisting of different loading rates of 0.5, 1, 2, and 3 wt.% of ${\mathrm{TiO}}_{2}$ nanoparticles were blended with 1 wt.% graphene, and the coating systems were developed accordingly via the solution intercalation method. First, using a cylindrical flask, 3 mL of butyl acetate (solvent) was measured and added into the beaker. Into the same beaker, the graphene/${\mathrm{TiO}}_{2}$ coating systems were measured and added accordingly. The mixture was then left to disperse for approximately 25 min with the use of a magnetic stirrer at 500 rpm. The blended mixture was then poured into the acrylic-epoxy polymer matrix and stirred for 15 min. Subsequently, IPDA and 1 wt.% PDMS was incorporated into the mixture and sonicated for another 15 min, followed by stirring the mixture for 15 min. This step was done to ensure that the mixture blended well together. The NCO curing agent was then added to the mixture and was manually stirred using a glass rod for approximately 2 min. The final blended coating mixture was applied on both sides of the cold-rolled mild steel substrates, having dimensions of 0.5 mm × 73 mm × 65 mm (thickness × width × length) and was left to cure at room temperature for five days. The steel substrates were abraded with a sandblaster according to the ASTM D609 standard, followed by acetone degreasing. An Elcometer 456 thickness gauge was used to measure the thickness of the films at several points for all the coatings. Table 1 shows the compositions of the prepared coating systems along with their corresponding notations and the average thickness of dry coating film after being applied on the substrate surface. Figure 1 illustrates the coated glass substrates for each corresponding coating system, respectively.

### 2.3. Characterization

#### 2.3.1. FTIR

FTIR analysis was performed to observe cross-linking and qualitative investigations. ATR-Nicolet iS10 Spectrometer (Thermo Fisher Scientific, Waltham, MA, USA) was utilized for this analysis in the range of 4000 to 400 cm^−1^ at a resolution of 4 cm^−1^ and the data was recorded after 32 scans.

#### 2.3.2. Thermal Analysis

By using Mettler Toledo TGA Q500 equipment that runs dynamically from 30 °C to 800 °C at a heating rate of 10 °C/min under nitrogen gas with a flow rate of 20 mL/min, the TGA tests were employed to evaluate the thermal stability of the hybrid nanocomposite coating systems. The TGA apparatus was purchased from Perkin Elmer, Pyris Diamond, USA and all the TGA thermograms were analyzed by the TARe software, TA Universal Analysis, Version 4.7A.

#### 2.3.3. Optical Transmittance

The UV-3101PC (Shimadzu, Kyoto, Japan) UV-vis spectrometer in transmission mode at a wavelength range of 200 nm to 800 nm on medium-speed scanning was employed to analyze the degree of transparency of the coatings coated on the glass substrates. The objective of this analysis was to evaluate the percentage changes in film transparency after the inclusion of ${\mathrm{TiO}}_{2}$ nanoparticles into the polymer matrix.

#### 2.3.4. Water Contact Angle (WCA) Analysis

The WCA test was carried out to evaluate the hydrophobic performance of the developed nanocomposite coatings by using an optical contact angle (OCA) 15EC instrument. At different locations, 5 droplets of 5 µL distilled water were dispensed on the surface of the specimens, and the images of static WCAs were captured, respectively. An average of three readings were recorded, and the highest value was reported with less than 2° as a measurement error.

#### 2.3.5. Coating Surface Adhesion Analysis

The effect of the graphene/${\mathrm{TiO}}_{2}$ on the coating’s adhesion was analyzed by the Cross-hatch test (CHT), following the ASTM D3359 B standard. A cross-hatch cutter (Elcometer 107) was gently placed on top of the coated surface. Mild pressure was applied to the coating layer, and the substrate surface was pulled steadily to form a series of parallel cuts approximately 20 mm long. A selective tape was then placed at the center of the lattice, and by using a typical pencil eraser, pressure was applied on the tape to ensure excellent adhesion of the tape onto the lattice. After two minutes, in a single action, the tape was pulled at an angle of 180° along the sample surface. All the adhesion images for each coating system were captured by a digital polarized microscope (Dino-Lite, AM413ZT).

#### 2.3.6. Electrochemical Impedance Spectroscopy (EIS)

The anticorrosion properties of the composite coatings were analyzed by EIS. Gamry potentiostat (model PC14G300, Warminster, PA, USA) was used, and the sinusoidal voltage amplitude was set at 10 mV. Herein, a three-electrode cell system was used for investigation for 90 days, and the electrolyte was 3.5 wt.% NaCl. The electrochemical cell was developed by placing a 2.0 cm inner diameter poly (vinyl chloride) (PVC) tube vertically on the coated substrate surface. On the other hand, the uncoated section was a working electrode, while the platinum as a counter electrode, and the saturated calomel electrode (SCE) was a reference electrode. In the frequency range of 100 kHz to 10 mHz, the EIS analysis was carried out after placing the electrochemical cell in a Faraday cage to minimize the noise.

#### 2.3.7. Surface Morphology

The FESEM was utilized to examine the surface morphology and the homogenous distribution of the nanoparticles within the polymer matrix. All the free film nanocomposite samples were coated with platinum by using a platinum sputter coater (Bio-Rad, Watford, England) in order to minimize the charging effects. The FEI Quanta 450 FEG with SDD EDS detector up to 5 KV as accelerating voltage in the presence of low vacuum was utilized to characterize the coating films.

## 3. Results and Discussion

### 3.1. Fourier-Transform Infrared Spectroscopy (FTIR)

**Figure 2:**
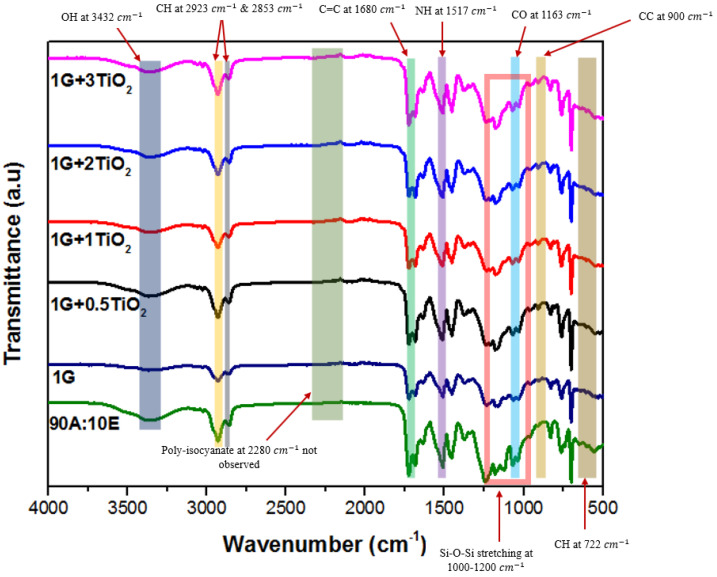
The FTIR spectra of the graphene/${\mathrm{TiO}}_{2}$ nanocomposite coating systems, along with the 1 wt.% graphene (1G) and the acrylic-epoxy (90A:10E) polymer nanocomposite coating samples, respectively.

The FTIR spectrometer was used to identify the appearance of the chemical functional groups of the graphene/${\mathrm{TiO}}_{2}$, 1 wt.% graphene (1G) and acrylic-epoxy (90A:10E) polymer nanocomposite coating samples from 4000 to 400 ${\mathrm{cm}}^{-1}$ (Figure 2). The bands at 1260 ${\mathrm{cm}}^{-1}$ and 1517 ${\mathrm{cm}}^{-1}$ were attributed to CN and NH stretching modes, respectively [47]. As a result of an effective curing process, the presence of the shifts in the peak of the functional groups indicated that the cross-linking had occurred between the resins, curing agent and graphene/${\mathrm{TiO}}_{2}$ nanoparticles, as a result of an effective curing process. For instance, in another study, it was reported that the identical peak for NCO group in the FTIR spectra was observed at 2280 ${\mathrm{cm}}^{-1}$ [47,48]. For instance, the formation of the NH bond occurred as the hardener cured the binder systems. As a result, the NCO band should not appear, but NH vibration should be observed in the FTIR spectra. Here, there was clear evidence that there were no NCO bands in the peak range for all the coating samples. Furthermore, the observable shift in the OH band in the 3300–3600$\;{\mathrm{cm}}^{-1}$ range confirmed the occurrence of blending of the resins. Moreover, in the approximate range of 1000–1090 ${\mathrm{cm}}^{-1}$, the peaks of ester were observed which belonged to the C-O-O stretching vibration [49]. In addition, the observed peak at approximately 3000$\;{\mathrm{cm}}^{-1}$ was attributed to the ${\mathrm{CH}}_{3}$ stretching band. Moreover, this was depicted by the peaks at approximately 1450 ${\mathrm{cm}}^{-1}$ and 1375 ${\mathrm{cm}}^{-1}$, indicating that the asymmetric and symmetric CH deformations had occurred in the ${\mathrm{CH}}_{3}$ bending [49]. Also, the peaks at approximately 1750 ${\mathrm{cm}}^{-1}$ and 1060 ${\mathrm{cm}}^{-1}$ signified the C=O and C-O stretching, which resulted due to the presence of carbonyl groups in the coating structure. In addition, the C=C bond was represented by the peak at 1680 ${\mathrm{cm}}^{-1}$ [49,50,51]. An intensified peak of approximately 1450 ${\mathrm{cm}}^{-1}$ indicated the presence of the ${\mathrm{TiO}}_{2}$ nanoparticles, and additionally, the Ti-O stretching band was also observed at approximately 880 ${\mathrm{cm}}^{-1}$ and 760 ${\mathrm{cm}}^{-1},$ respectively [49,50,51]. Furthermore, the broad peak observed at approximately 3432 ${\mathrm{cm}}^{-1}$ and 1615 ${\mathrm{cm}}^{-1}$ were assigned to the OH groups, which indicated the presence of water in the compound. The existence peaks at 2923 ${\mathrm{cm}}^{-1}$ and 2853 ${\mathrm{cm}}^{-1}$ represented the CH stretching. Finally, the presence of the peaks in the range of 1000–1200 ${\mathrm{cm}}^{-1}$ were attributed to the Si-O-Si bands of PDMS in the hybrid coatings, which indicated that this band overlapped with the C-O stretching band [49,52]. Here, the changes were difficult to be observed due to the presence of a wide band in this range. Hence, the confirmation of the graphene/${\mathrm{TiO}}_{2}$ nanocomposite coating structure was validated by the FTIR analysis.

### 3.2. Thermal Analysis

**Figure 3:**
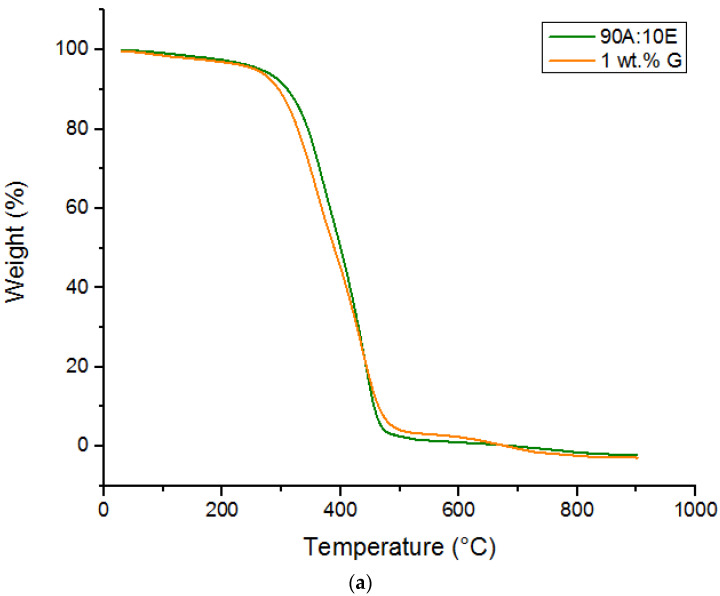

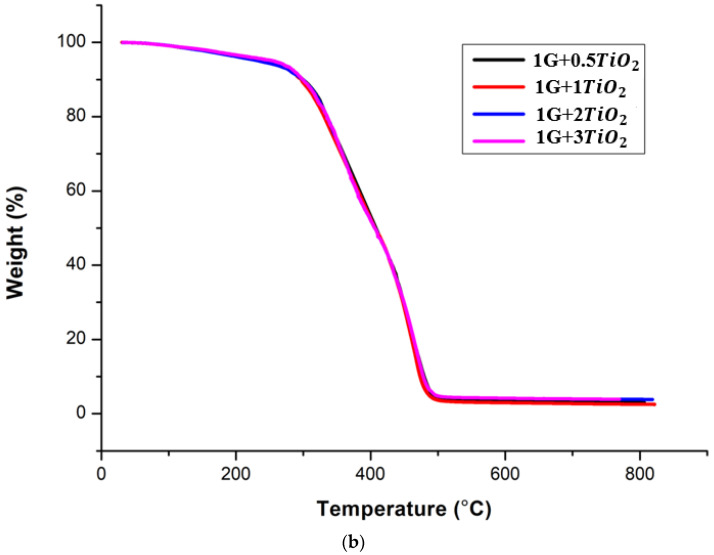
TGA curves of (**a**) 90A:10E (wt.%) polymer binder and 1 wt.% graphene (1G) based polymer coating and (**b**) the graphene/${\mathrm{TiO}}_{2}$ based nanocomposite coatings, respectively.

Figure 3a,b illustrates the TGA curves for the acrylic-epoxy binder coating (90A:10E), 1 wt.% graphene (1G) based polymer coating and all the graphene/${\mathrm{TiO}}_{2}$ nanocomposite coating samples for heating temperatures ranging from 30 °C to 900 °C. Tabulated in Table 2 is the temperature where 50% weight loss ($T_{50\mathrm{wt}\%}$) occurred, and the residue yields at 700 °C along with the initial degradation temperature (IDT) values. In addition, it was observed that two-step decomposition had occurred for all the investigated coating samples. For all the coating systems, several stages of decomposition were observed. At approximately 100 °C range, there was a reduction in weight percentage which could be due to the evaporation of water moisture and some unevaporated solvents present in the coatings. This finding was validated as per the datasheet obtained from the manufacture, whereby the boiling temperature range was 124–128 °C. Here, a linear rate up to 290 °C was exhibited by the loss of mass. The second stage corresponded to the degradation of the NCO curing agent that occurred at the temperature of 290–400 °C. This was also in accordance with its datasheet, which reported that the boiling point of NCO resin was at 280 °C. Subsequently, between 390–490 °C, the third stage corresponded to the extensive breakdown of the chemical bonds in the epoxy and acrylic resin network, which had a boiling point of approximately 370 °C. The final stage occurred when the temperature exceeded 500 °C, which represented the loss of the PDMS, which is known to have an ignition temperature of approximately 510 °C. However, in this section, only the role of ${\mathrm{TiO}}_{2}$ nanoparticles at different loading ratios (0.5–3 wt.%) were reported in order to understand the significance of ${\mathrm{TiO}}_{2}$ nanoparticles in altering the thermal stability of the graphene/${\mathrm{TiO}}_{2}$ based hybrid nanocomposite coating systems. Alternatively, as shown in Table 2, the $T_{50\mathrm{wt}\%}$ values for all the coating systems were slightly enhanced as compared to the reference coatings 90A:10E and 1 wt.% graphene sample, respectively. The degradation has been delayed with the addition of graphene and TiO_2_ which is evidenced by the increase in the residue values. In other words, over the temperature range, some changes at each degradation occurred with the increment of the loading rates of ${\mathrm{TiO}}_{2}$ nanoparticles. Moreover, it is important to note that none of the loading ratios of the ${\mathrm{TiO}}_{2}$ nanoparticles resulted in any significant change to the IDT and $T_{50\mathrm{wt}\%}$ values, for instance, as compared to the 1 wt.% G coating system, the graphene/${\mathrm{TiO}}_{2}$ coating systems exhibited values where the maximum difference was less than 5 °C. Moreover, when the 0.5 wt.% and 1 wt.% of ${\mathrm{TiO}}_{2}$ nanoparticles were employed into the 1 wt.% G, it was observed that $T_{50\mathrm{wt}\%}$ values had slightly increased from 405.16 °C to 406.71 °C. For the IDT values, it was observed that these values varied for all the coating systems. For instance, at 0.5 wt.% ${\mathrm{TiO}}_{2}$, the IDT value was reported at 282.76 °C and had slightly decreased to 280.43 °C with 1 wt.%. ${\mathrm{TiO}}_{2}$. Similarly, at 2 wt.% ${\mathrm{TiO}}_{2}$, the IDT values had increased to 290.31 °C followed by a slight drop to 288.57 °C when the highest loading ratio of ${\mathrm{TiO}}_{2}$ (3 wt.%) was incorporated. Overall, both IDT and $T_{50\mathrm{wt}\%}$ values exhibited by these coatings systems were higher compared to the 1 wt.% G. The higher thermal stability was further indicated due to the presence if Si-O-Si bonds in the PDMS structure, thus, being responsible for the thermal stability enhancement of the coatings [53]. However, the inclusion of ${\mathrm{TiO}}_{2}$ nanoparticles within the graphene/epoxy-acrylic hybrid polymeric matrix showed a mixture of slight increment and decrement trends for both IDT and $T_{50\mathrm{wt}\%}$ values, which indicated a slight decrease in the thermal stability of the developed graphene/${\mathrm{TiO}}_{2}$-based hybrid nanocomposite coatings. Similar findings were previously reported with regard to this phenomenon, whereby the presence of these nanoparticles resulted in the reduction of the mobility of the chains in the polymeric matrix near the surfaces of the dispersed nanoparticles [54,55]. With respect to the curing state of all the developed coating systems, the 1G+1${\mathrm{TiO}}_{2}$ coating system exhibited the highest $T_{50\mathrm{wt}\%}$ value and the best overall performance in terms of the enhancement of the thermal stability.

### 3.3. Optical Transmittance

**Figure 4:**
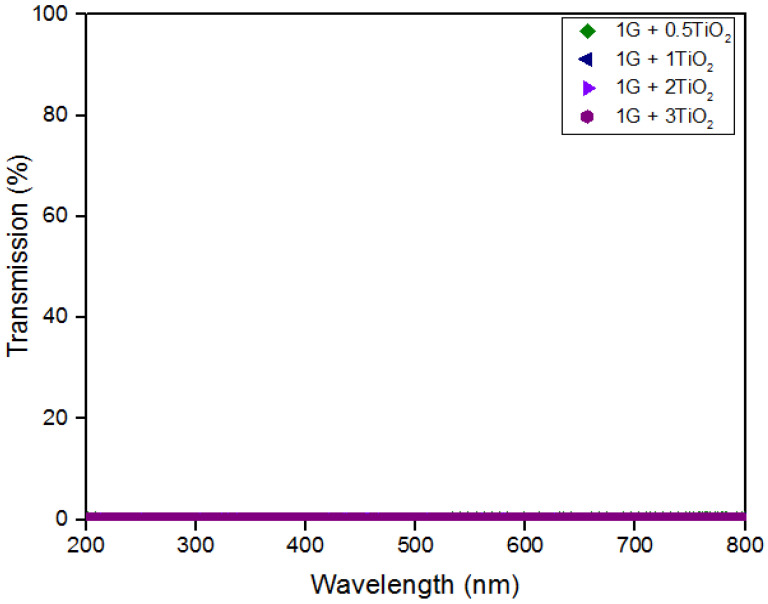
UV transparency curves for the graphene/${\mathrm{TiO}}_{2}$ coating samples.

In this study, the UV-Vis spectroscopy was used to evaluate the optical properties of the graphene/${\mathrm{TiO}}_{2}$ nanocomposite coating films. Figure 4 illustrates the UV-vis transmittance spectra for all the graphene/${\mathrm{TiO}}_{2}$ coating systems in the visible wavelength range of 200 to 800 nm. Furthermore, there is clear evidence that the transmittance percentage did not show any significant change with the increment of the loading ratio of the ${\mathrm{TiO}}_{2}$ nanoparticles. As reported previously, with the inclusion of just 1% graphene into the polymeric matrix, the coatings were able to effectively block the incoming radiation [56]. Alternatively, it is known that a thin layer of ${\mathrm{TiO}}_{2}$ has a high transparency, between 70–100%, particularly in the visible wavelength range of 600 to 800 nm [57]. However, in the previous years, researchers also reported that the effect of adding graphene and GO on the optical band gap of ${\mathrm{TiO}}_{2}$ has been limitedly investigated [57]. Despite the fact that a higher ${\mathrm{TiO}}_{2}$ content incorporated within the polymeric matrix will result in an improved transparency in the visible range, however, in this case, the inclusion of the ${\mathrm{TiO}}_{2}$ nanoparticles did not affect the optical properties of the coating films [58]. In other words, zero percent transmittance were shown throughout the visible range. This is due to the high coating thickness, which resulted in the agglomeration of the ${\mathrm{TiO}}_{2}$ nanoparticles. Moreover, Huang and Hsieh et al. stated that the reduced transmission intensity in a composite coating sample is influenced by the size and the dispersion of the nanoparticles in the system [59]. From Figure 1, it can be seen that the coatings on the glass substrate were thick and darker in color (no transparency), thus indicating that visible light will not be able to penetrate through the coatings. These results were in strong agreement with this research work based on graphene coatings exhibiting low optical transmittance [56,60].

### 3.4. Water Contact Angle (WCA) Measurements

**Figure 5:**
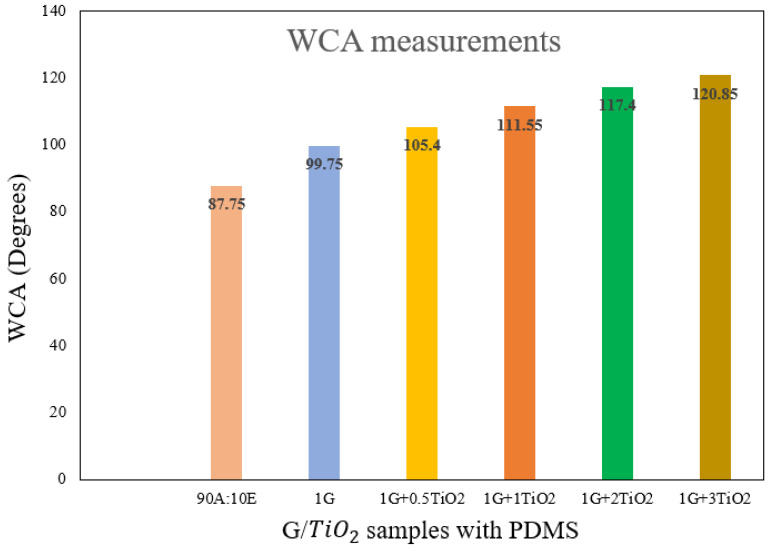
WCA values (degrees) for all the coating samples with the incorporation of PDMS.

**Figure 6:**
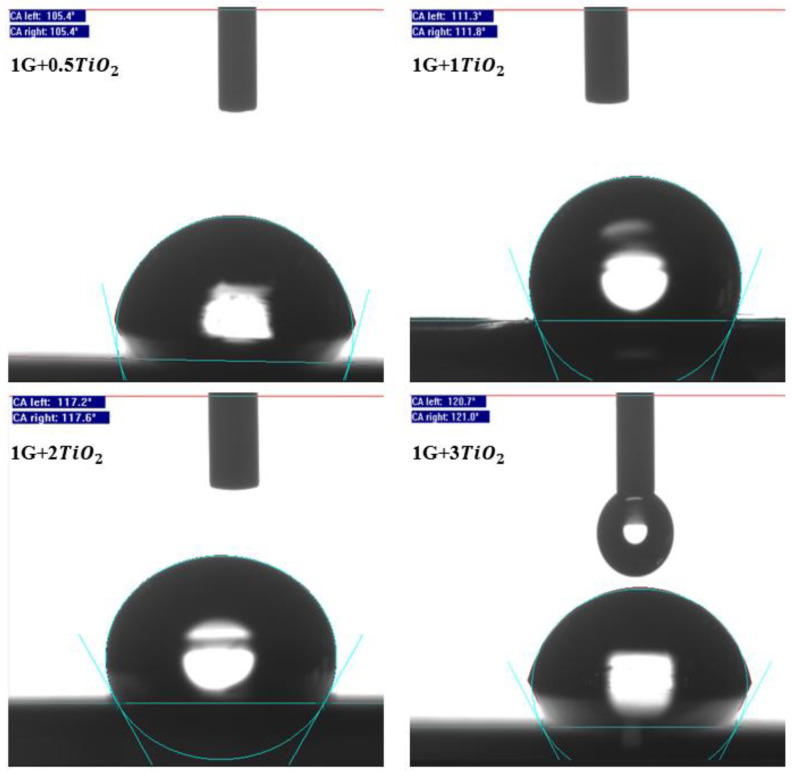
The WCA surface characterization images for all the graphene/${\mathrm{TiO}}_{2}$ coatings samples with the incorporation of PDMS.

As illustrated in Figure 5, all the graphene/${\mathrm{TiO}}_{2}$ nanocomposite coating samples achieved hydrophobic properties, ranging from 105.4° to 120.85° compared to the polymer matrix (90A:10E) and 1 wt.% graphene (1G) coating samples, respectively. For instance, without the addition of graphene and ${\mathrm{TiO}}_{2}$ nanoparticles, the 90A:10E polymeric coating sample exhibited a WCA of 87.75°. However, by incorporating 1 wt.% of graphene into the polymeric matrix, the WCA was observed to significantly enhance to 99.75° [61]. Interestingly and as expected, the inclusion of the combined graphene/${\mathrm{TiO}}_{2}$ nanoparticles with different concentrations of ${\mathrm{TiO}}_{2}$ nanoparticles significantly enhanced the surface roughness of the coatings, exhibiting the highest CA value of 120.85°. In addition, this occurrence suggests that the inclusion of graphene and ${\mathrm{TiO}}_{2}$ nanoparticles resulted in the enhancement of the surface roughness of the coating and concomitantly improved the non-wettability of the surface. Hence, the exhibited results are in concordance with the literature, which clearly states that the surface roughness and the chemical composition of the surface influence the hydrophobic and super-hydrophobic properties, respectively [49]. Furthermore, due to the presence of micro/nano structures on the graphene/${\mathrm{TiO}}_{2}$ surface, this resulted the surface roughness to entrap a sufficient air layer on the surface. Recently, graphene-based acrylic-epoxy nanocomposite coatings were fabricated by incorporating different loading rates of graphene nanoparticles (0.5–7 wt.%) and its surface wettability were evaluated [61]. Here, it was observed that the 1 wt.% graphene coating sample exhibited the highest WCA of approximately 100°. However, at higher loading rates of graphene nanoparticles (>5 wt.%), agglomeration of the nanoparticles was observed, thus, indicating that the film defects had formed and resulting in the WCA decreasing to 83° [61]. Figure 6 illustrates the WCA values that were obtained from the WCA surface characterization for all the coating samples, respectively.

In addition, it can be clearly seen that the concentration of ${\mathrm{TiO}}_{2}$ nanoparticles increased from 0.5% to 3%, having the graphene content to be constant at 1%, the CA of the coating surface increased exponentially. As mentioned above, it has been revealed that a higher CA was exhibited by the graphene/${\mathrm{TiO}}_{2}$ coatings compared to the graphene coatings. In other words, these results provided evidence of the synergistic effect whereby the combined graphene/${\mathrm{TiO}}_{2}$ promoted hydrophobic properties. Also, an appropriate interspacing between them was conferred by the mixture of these nanoparticles. Additionally, the incorporation of PDMS into these coatings lowered the surface energy of the modified surface, thus, resulting in the wettability of these coated surfaces being lowered. As mentioned previously, the cross-linking induced by the PDMS helps to enhance the durability and elasticity of the coated film [49].

Interestingly, it is noteworthy to mention that at the highest ${\mathrm{TiO}}_{2}$ loading of 3%, the highest WCA of 120.85° was achieved. On the other hand, the inclusion of 0.5%, 1%, and 2% of ${\mathrm{TiO}}_{2}$ loading had exhibited a WCA value of 105.4°, 111.55°, and 117.4°, respectively, thus indicating that the presence of ${\mathrm{TiO}}_{2}$ in the coating created a rough texture on the coating surface and further enhanced the surface roughness of the coating. In addition, with the increment of the ${\mathrm{TiO}}_{2}$ content, the surface roughness was further increased by these nanoparticles by reducing the chemical inhomogeneity while maintaining the structure of the surface. Hence, this condition resulted in greater hydrophobicity [57]. Furthermore, the inclusion of a 1 wt.% loading rate of graphene was mainly selected because the higher content of graphene nanoparticles resulted in the creation of an uneven surface with large spacings, thus, lowering the WCA [62]. To support this finding, Wu et al. reported that a WCA value of 164.21° was achieved with a graphene-to-${\mathrm{TiO}}_{2}$ ratio of 1:9 [49]. Alternatively, when the ratio of graphene-to-${\mathrm{TiO}}_{2}$ was equal, the WCA value was reported to decrease to approximately 161°, and with the ratio of graphene-to-${\mathrm{TiO}}_{2}$ of 9:1, the WCA was observed to significantly decrease to approximately 100° [49]. In short, both graphene and ${\mathrm{TiO}}_{2}$ nanoparticles exert a synergistic effect to produce a surface with hydrophobic and super-hydrophobic properties.

### 3.5. Cross-Hatch Test (CHT) Analysis

**Figure 7:**
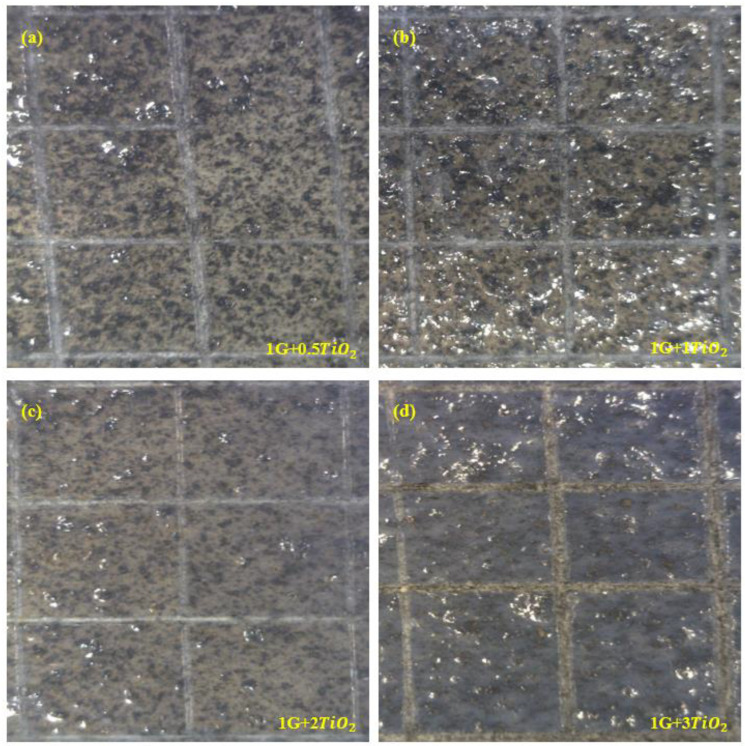
Representations of the CHT analysis performed on all the graphene/${\mathrm{TiO}}_{2}$ nanocomposite coating samples (**a**–**d**).

The adhesion of the coating film to the metal substrate is considered to be an important factor in order to develop an intact coating system with excellent overall performance. In this analysis, the adhesion properties of the graphene/${\mathrm{TiO}}_{2}$ hybrid nanocomposite coating systems were investigated by the CHT. Figure 7a–d illustrates the cross-cut images of all the samples that were performed according to the ASTM D3359 method B standard. The inclusion of ${\mathrm{TiO}}_{2}$ within the hybrid polymeric matrix in different loading rates of 0.5 wt.%, 1 wt.% and 2 wt.% did not result in any significant changes in the adhesion behavior of the coating films. These coatings have been classified as 5B adhesion grade, which implied that there were cuts with smooth edges without any loss of adhesion or detachment of the lattice squares, as illustrated in Figure 7a–c. Moreover, the proper dispersion of the ${\mathrm{TiO}}_{2}$ nanoparticles within the polymeric matrix, and the good curing state of the applied coating films were further confirmed from these observations. On the other hand, as, in this case, the loading ratio of ${\mathrm{TiO}}_{2}$ increased by 3 wt.%, it was observed that the adhesion properties slightly decreased. As illustrated in Figure 7d, the detachment of the flakes of the coating at the intersections of the cut was observed to be lesser than 5%. Hence, the coating containing 3% of ${\mathrm{TiO}}_{2}$ was classified as 4B adhesion grade. As mentioned earlier, this phenomenon could be due to the tendency of the nanoparticles to agglomerate, thus affecting the bulk properties of the coating film rather than the surface properties. Overall, all the graphene/${\mathrm{TiO}}_{2}$ hybrid nanocomposite coating systems exhibited excellent adhesion behavior of the coatings, receiving 5B and 4B adhesion grades according to the ASTM standard.

### 3.6. Electrochemical Impedance Spectroscopy Evaluations

**Figure 8:**
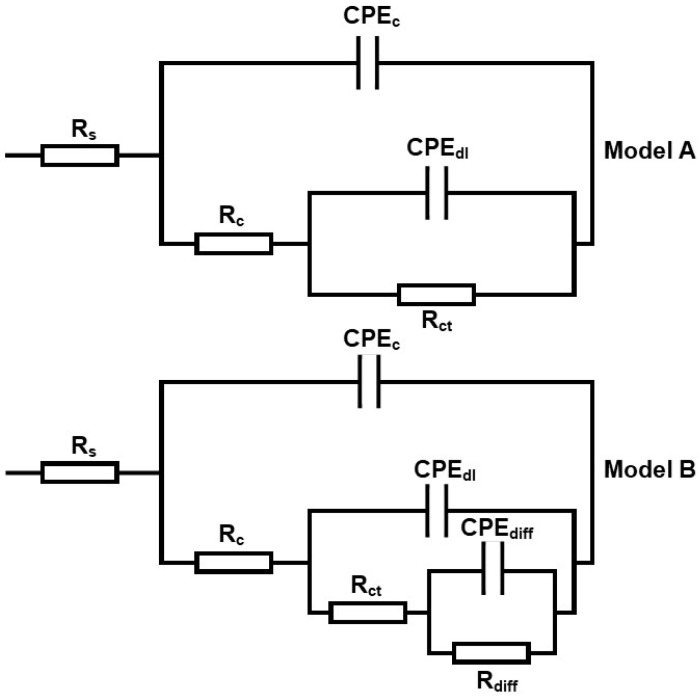
Schematic diagram of the equivalent circuits used for the fitting of impedance plots.

**Figure 9:**
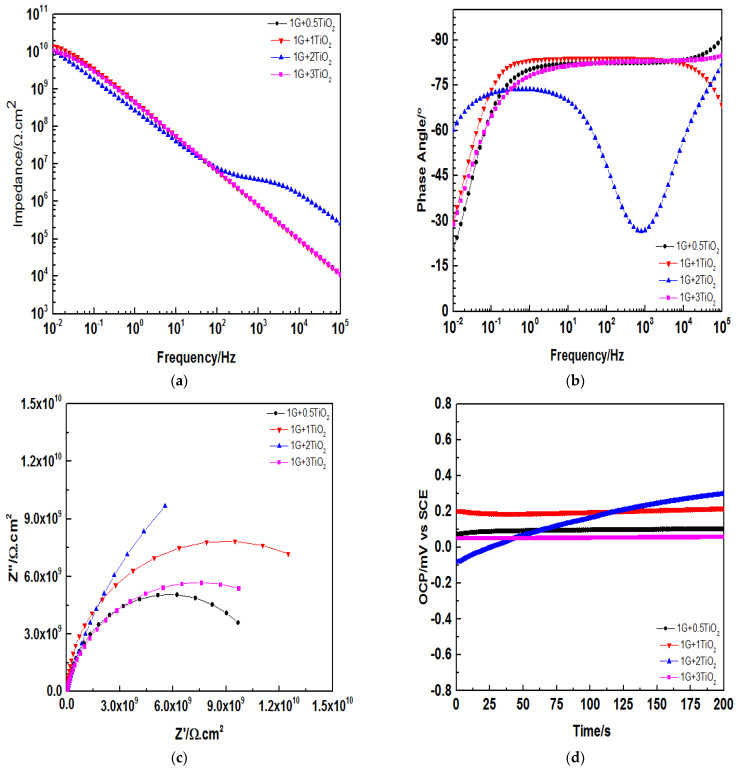
The representations of (**a**) Bode plot, (**b**) Phase angle plot, (**c**) Nyquist plot, and (**d**) OCP for all the graphene/${\mathrm{TiO}}_{2}$ nanocomposite coating samples modified with PDMS after one day of immersion.

**Figure 10:**
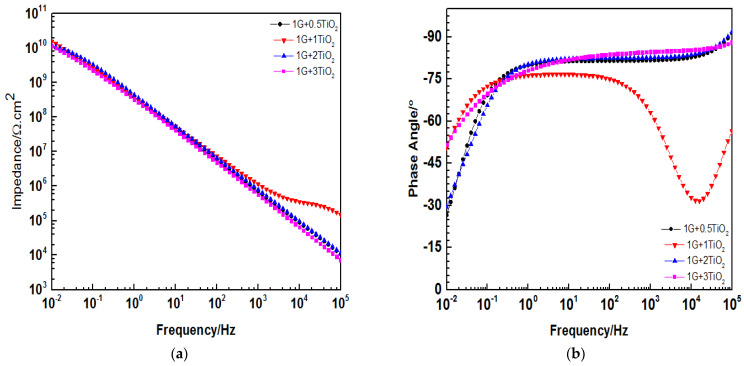

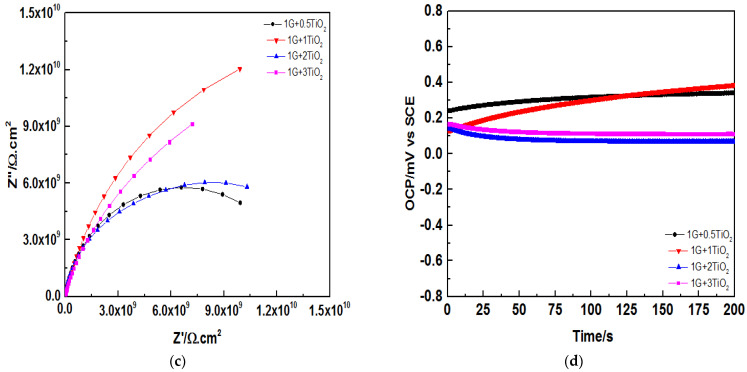
(**a**) Bode plot, (**b**) Phase angle plot, (**c**) Nyquist plot, and (**d**) OCP for all the graphene/${\mathrm{TiO}}_{2}$ nanocomposite coating samples modified with PDMS after 30 days of immersion.

**Figure 11:**
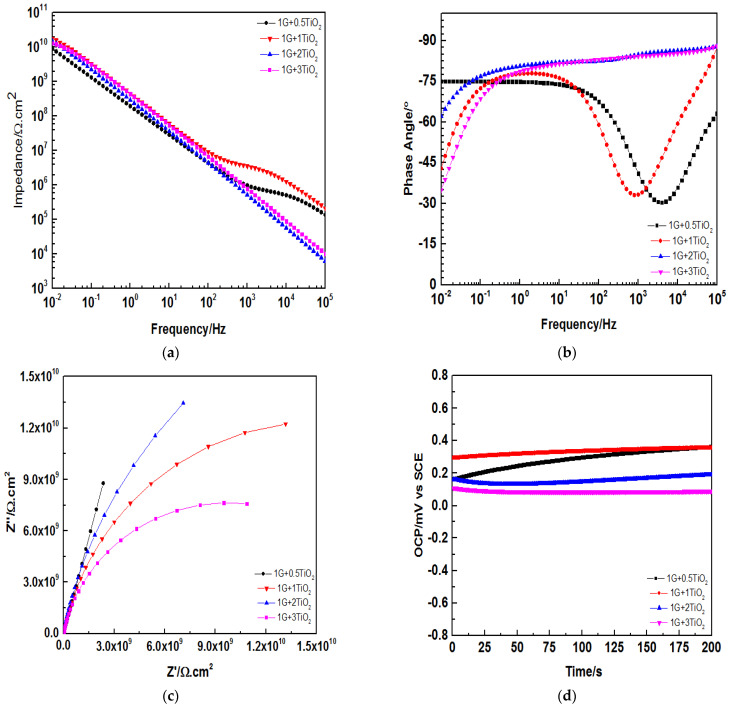
(**a**) Bode plot, (**b**) Phase angle plot, (**c**) Nyquist plot, and (**d**) OCP for all the graphene/${\mathrm{TiO}}_{2}$ nanocomposite coating samples modified with PDMS after 60 days of immersion.

**Figure 12:**
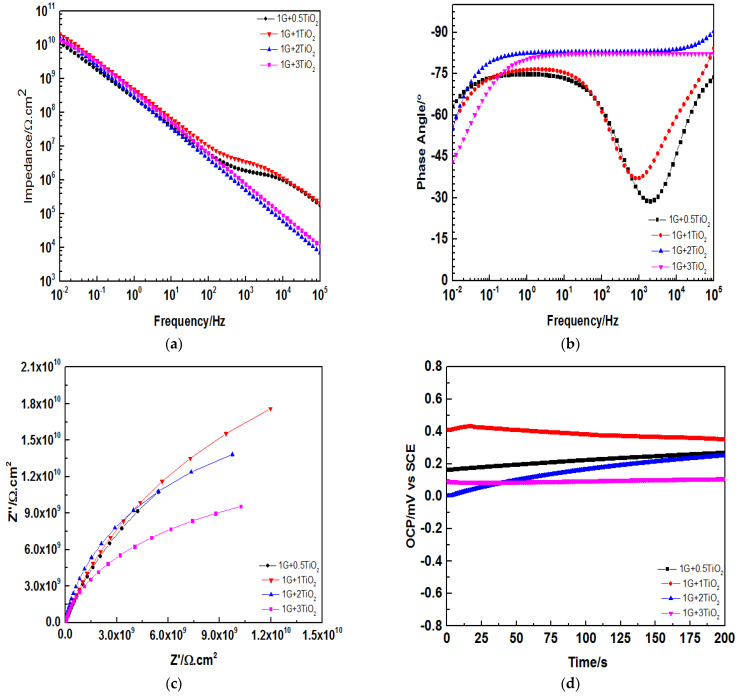
(**a**) Bode plot, (**b**) Phase angle plot, (**c**) Nyquist plot, and (**d**) OCP for all the graphene/${\mathrm{TiO}}_{2}$ nanocomposite coating samples modified with PDMS after 90 days of immersion.

The corrosion resistance of the graphene-based hybrid polymer nanocomposite coating systems with different inclusion rates of ${\mathrm{TiO}}_{2}$ nanoparticles (0.5–3 wt.%) were investigated by the EIS measurements after exposing the coated steel substrates to 3.5 wt.% NaCl solution. Furthermore, on the first day, 30th day, 60th day and 90th day, the impedance plots after different immersion periods were fitted using two models of equivalent circuits, as depicted in Figure 8. The obtained results were expressed graphically using Bode plots, as illustrated in Figure 9, Figure 10, Figure 11 and Figure 12, respectively. In general, the corrosion of the coating process of the coating is divided into several periods. Firstly, the solution penetrates into the interior of the coating via the surface micropores, particularly in the early corrosion solution. Over a period of immersion time, the characteristics of a two-time constant in the impedance spectrum indicate that the corrosion has entered the mid-period, whereby the corrosive ions have started to penetrate the coating surface. However, the coating still has a protective effect despite the solution being diffused into the interface between the coating and the substrate. Finally, in a short period of time, the corrosion process will move on to the next period, thus, exhibiting low impedance values, which indicate the poor anticorrosive performance of the coating. Hence, in this approach, only the influence of ${\mathrm{TiO}}_{2}$ nanoparticles with different loading ratios into the polymeric matrix will be discussed.

Due to the good corrosion resistance of ${\mathrm{TiO}}_{2}$, from Figure 9a, the Bode plot revealed that all the developed hybrid coating systems demonstrated high (${\left|Z\right|}_{0.01\;\mathrm{Hz}}$) values after one day of immersion, exceeding $10^{10}$ Ω$\;{\mathrm{cm}}^{2}$ and consisting of a straight line with a gradient of −1. Here, these exhibited (${\left|Z\right|}_{0.01\;\mathrm{Hz}}$) values were observed to be higher compared to the 1 wt.% G coating system. In other words, the (${\left|Z\right|}_{0.01\;\mathrm{Hz}}$) values here were observed to increase by one magnitude of order compared to the 1 wt.% G coating system, which exhibited (${\left|Z\right|}_{0.01\;\mathrm{Hz}}$) values ranging between $10^{9}$ Ω$\;{\mathrm{cm}}^{2}$ to $10^{10}$ Ω$\;{\mathrm{cm}}^{2}$. Among these hybrid coating systems, it was observed that the 1G+1${\mathrm{TiO}}_{2}$ coating system exhibited the highest ${\left|Z\right|}_{0.01\;\mathrm{Hz}}$ value. On an important note, as shown in Figure 9a, after one day of immersion, no obvious bend in the low-frequency region was observed, thus, indicating that the inclusion of ${\mathrm{TiO}}_{2}$ nanoparticles into the polymeric matrix played an essential role in enhancing the corrosion protection performance of the coatings. Figure 9b, the phase angles for all the coating systems were observed to be close to 85° at high frequency. Figure 9c illustrates one capacitive loop that was exhibited in the Nyquist plot, thus, clearly indicating the efficiency of these coating systems to restrict any electrolyte penetration and complete isolation of the metal surface from the corrosion medium surrounding. At this stage of immersion, the EIS diagrams were fitted using model A of the equivalent circuit, as there was no diffusion of the electrolyte ions within the coating films. Gowri et al. reported that the obtained maximum corrosion-resistant specimen exhibited a rise in the potential to a more positive value, and the remaining samples exhibited constant potential or became more negative. Figure 9d illustrates the OCP for all the coating systems after one day of immersion. Here, similar trends were observed whereby all the coating systems exhibited a more positive OCP value at 200s. From all the above findings, it was proven that the 1G+1${\mathrm{TiO}}_{2}$ coating system exhibited the best results compared to other coating systems after 24 h of immersion.

As the exposure time elapsed, no significant change or bend was observed in the slope of the Bode plot at low-frequency region for all the coating systems after 30 days of immersion, whereby the ${\left|Z\right|}_{0.01\;\mathrm{Hz}}$ values exceeded $10^{10}$ Ω.${\mathrm{cm}}^{2}$, as illustrated in Figure 10a. All these coating films demonstrated good barrier properties and had a strong ability to withstand corrosion action, as discussed in the previous results. Also, these coatings were intact, and no delamination had occurred. Moreover, by referring to the Bode plot diagram, the 1G+1${\mathrm{TiO}}_{2}$ coating system exhibited the highest ${\left|Z\right|}_{0.01\;\mathrm{Hz}}$ value after 30 days of immersion. Hence, this occurrence provided evidence that the inclusion of 1 wt.% ${\mathrm{TiO}}_{2}$ nanoparticles was sufficient to fill all the pores in the polymeric matrix. In addition, at high frequency, Figure 10b depicts the phase angles for all the coatings at approximately 90°. However, for 1G+1${\mathrm{TiO}}_{2}$, the phase angle exhibited was slightly lower (60°), and this could be attributed to the coating thickness.

Figure 10c illustrates the Nyquist plot for all the coating systems. It was observed that one capacitive loop was exhibited by these coating systems, thus, showing the character of one time constant. Therefore, this observation indicated that these coating systems provided good corrosion barrier protection to the metal substrate, whereby the 1 wt.% G exhibited the best results among all the coating systems. Moreover, the excellent dispersibility of the blended graphene and ${\mathrm{TiO}}_{2}$ nanoparticles within the polymeric matrix resulted in the improvement of the efficiency of the corrosion-resistant medium, hence enhancing the corrosion resistance of all the coating films. The results of these coating systems were fitted with the Model A. Moreover, Figure 10d illustrates the OCPs for all the coating systems after 30 days of immersion. Again, all the coating systems exhibited positive OCP values. Overall, the best overall corrosion protection performance was exhibited by the 1G+1${\mathrm{TiO}}_{2}$ coating system.

Interestingly, the ${\left|Z\right|}_{0.01\;\mathrm{Hz}}$ value of above $10^{10}$ Ω ${\mathrm{cm}}^{2}$ was still shown by all the coating systems even after 60 days of immersion, whereby the highest ${\left|Z\right|}_{0.01\;\mathrm{Hz}}$ value was demonstrated by the 1G+1${\mathrm{TiO}}_{2}$ coating system, as depicted in Figure 11a. Furthermore, no significant bend was observed at the lower frequency region for all the coating systems, and the Bode plot data was fitted with model A. As illustrated in Figure 11b, at high frequency, all the coating systems except for 1G+0.5${\mathrm{TiO}}_{2}$ coating system exhibited a phase angle close to 90°. From these findings, the higher exhibited phase angles were due to the ability of the nanofillers to completely fill the coating porosity by dispersing uniformly within the polymeric matrix, hence, resulting in a more compact and effective coating.

Figure 11c illustrates the Nyquist plots for all the coatings. Similar to the first and 30th days, all the coating systems exhibited one capacitive loop and showed the character of one time constant. In short, no significant changes were observed in the Bode and Nyquist plots for all the coating over the immersion time period from the first to 60 days. In addition, Figure 11d illustrates the OCPs for all the coating after 60 days of exposure. Similar to the previous results, it was observed that all the coating systems exhibited positive OCP values. Hence, it can be concluded that the 1G+1${\mathrm{TiO}}_{2}$ coating system exhibited the highest OCP value and demonstrated the best corrosion barrier performance.

After 90 days of immersion, as depicted in Figure 12a, it was observed that 1G+1${\mathrm{TiO}}_{2}$ coating system still maintained the highest ${\left|Z\right|}_{0.01\;\mathrm{Hz}}$ of $10^{10}$ Ω ${\mathrm{cm}}^{2}$ at low-frequency range compared to the other coating systems, which also exhibited similar results. Here, it was concluded that the most pronounced effect of graphene and ${\mathrm{TiO}}_{2}$ nanoparticles was revealed when 1 wt.% was added into the polymeric matrix. On an important note, no further enhancement was observed with the increment of the loading ratios of ${\mathrm{TiO}}_{2}$ nanoparticles. This was due to the tendency of the nanoparticles to aggregate and agglomerate at higher loading ratios, as described in the previous results. In addition, as illustrated in Figure 12b, with the increment of the loading ratios of ${\mathrm{TiO}}_{2}$ nanoparticles, the phase angles were observed to increase. For instance, at loading ratios of 1, 2, and 3, wt.% of ${\mathrm{TiO}}_{2}$ nanoparticles, the phase angle at high frequency region was observed to range between 80–90°. This shows that the increment of ${\mathrm{TiO}}_{2}$ nanoparticles up to 3 wt.% into the polymeric matrix enhanced the phase angle and the coating properties and did not result in any improper dispersion of the coating. Furthermore, Figure 12c illustrated that after 90 days of immersion, all the coating systems exhibited one capacitive loop and showed the character of one time constant, thus, revealing that all the coating films continued to act as perfect capacitors even after such long-term exposure. At this stage of immersion, it was noteworthy to mention that the EIS results could still be fitted with model A. Finally, the OCPs for all the coatings after 90 days of immersion were illustrated in Figure 12d. Here, all the coating systems exhibited positive OCP values, whereby the 1G+1${\mathrm{TiO}}_{2}$ coating system exhibited the highest OCP of approximately 0.40 mV at 200s. Finally, Table 3 illustrates all the recorded ${\left|Z\right|}_{0.01\;\mathrm{Hz}}$ values for all the graphene/${\mathrm{TiO}}_{2}$ coating systems from the first day up to 90 days of immersion, which were in complete agreement with the explanations provided in this section. In conclusion, the incorporation of graphene nanofillers and ${\mathrm{TiO}}_{2}$ nanoparticles as inhibitors lead to remarkable enhancements in the anticorrosion properties of the developed coating systems via zigzagging of the diffusion pathways, which forces the corrosive agents to travel a longer distance to reach the surface of the substrate.

**Figure 13:**
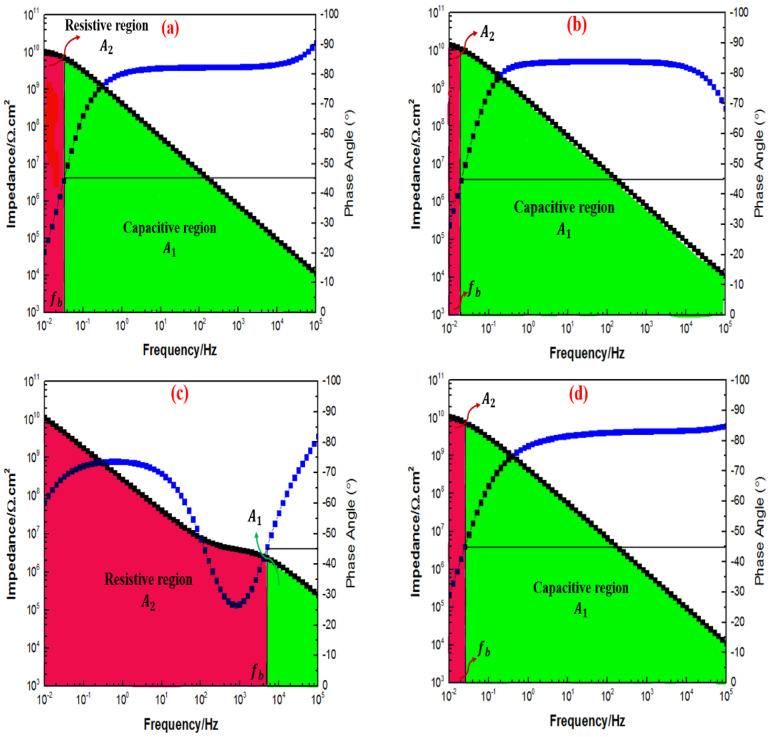
Bode plot with $f_{b}$ determination for all the graphene-based coating systems containing 0.5%, 1%, 2%, and 3% ${\mathrm{TiO}}_{2}$ nanoparticles (in the sequence of (**a**–**d**)) after one day of immersion with the corresponding capacitive ($A_{1}$) and resistive ($A_{2}$) regions, respectively.

**Figure 14:**
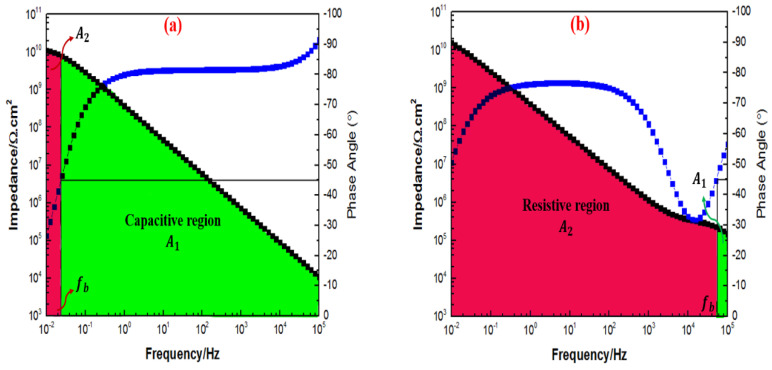

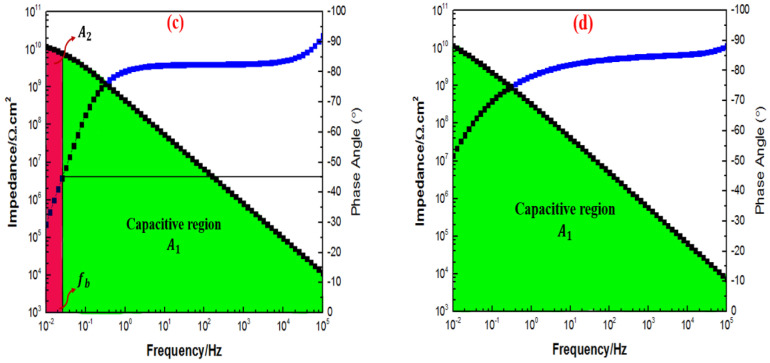
Bode plot with $f_{b}$ determination for all the graphene-based coating systems containing 0.5%, 1%, 2%, and 3% ${\mathrm{TiO}}_{2}$ nanoparticles (in the sequence of (**a**–**d**)) after 30 days of immersion with the corresponding capacitive ($A_{1}$) and resistive ($A_{2}$) regions, respectively.

**Figure 15:**
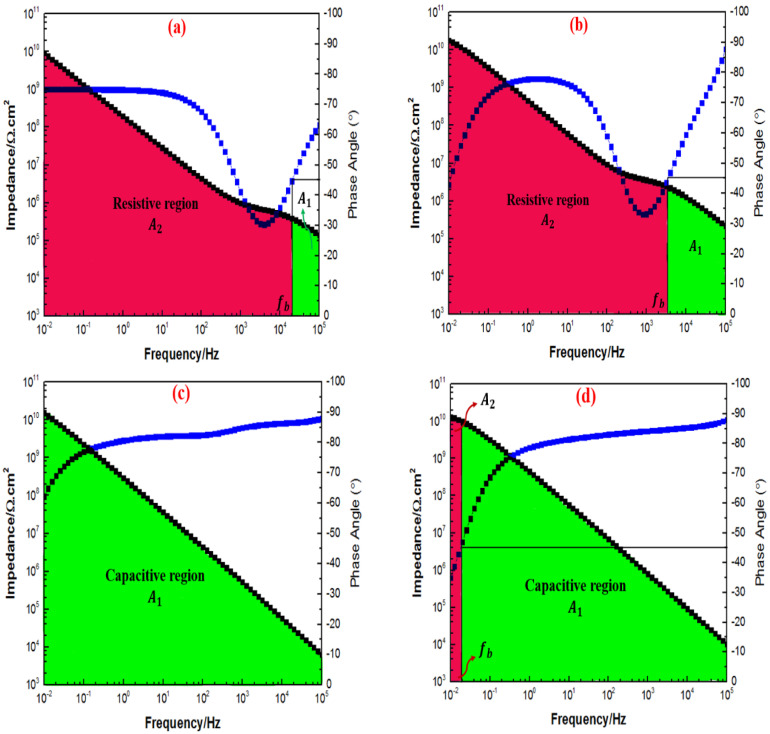
Bode plot with $f_{b}$ determination for all the graphene-based coating systems containing 0.5%, 1%, 2%, and 3% ${\mathrm{TiO}}_{2}$ nanoparticles (in the sequence of (**a**–**d**)) after 60 days of immersion with the corresponding capacitive ($A_{1}$) and resistive ($A_{2}$) regions, respectively.

**Figure 16:**
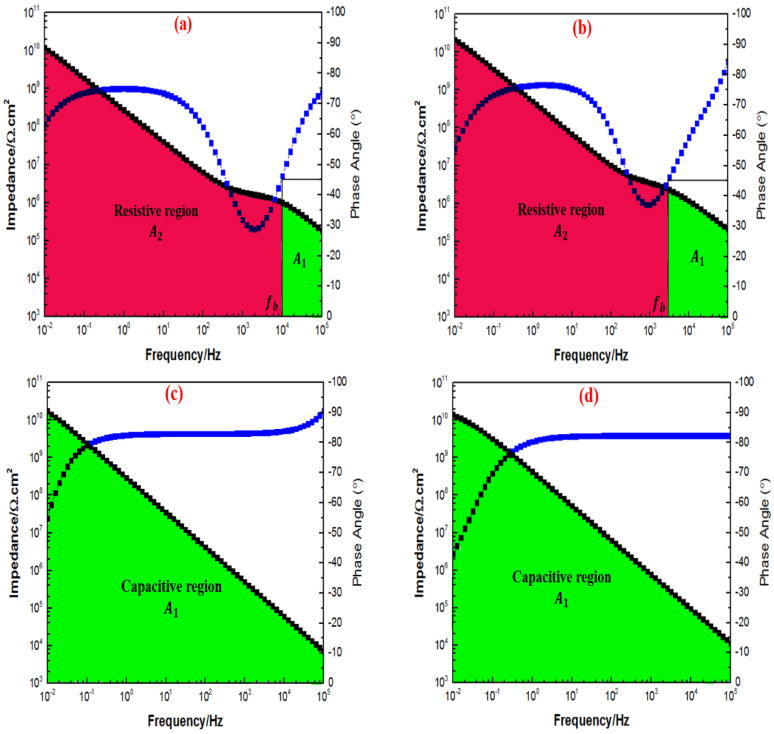
Bode plot with $f_{b}$ determination for all the graphene-based coating systems containing 0.5%, 1%, 2%, and 3% ${\mathrm{TiO}}_{2}$ nanoparticles (in the sequence of (**a**–**d**)) after 90 days of immersion with the corresponding capacitive ($A_{1}$) and resistive ($A_{2}$) regions, respectively.

Figure 13 illustrates the capacitive and resistive regions that have been developed from the $f_{b}$ borderline in order to evaluate the electrochemically active area of the coating systems after one day of immersion. Figure 13a–d, Figure 14a–d, Figure 15a–d and Figure 16a–d, respectively, illustrate the Bode plot with $f_{b}$ determination for all the coating systems that contained 0.5%, 1%, 2%, and 3% loading ratios of ${\mathrm{TiO}}_{2}$ nanoparticles after 1, 30, 60, and 90 days of immersion. From Figure 13, it was observed that both resistive and capacitive areas under the Bode plot were present after one day of immersion, where a majority of the areas were dominated by the capacitive region for all the coating systems, except for 1G+2${\mathrm{TiO}}_{2}$. Secondly, after 24 h, the increase in the size of the capacitive region over the resistive region was also observed, as depicted in Figure 13a–b. Here, a lower $f_{b}$ value was recorded, whereby smaller resistive regions at the lower frequency range and large capacitive regions were observed. Next, in Figure 13c, only the 1G+2${\mathrm{TiO}}_{2}$ coating system showed that the resistive region was more dominant compared to the capacitive region after one day of immersion, but Figure 13d illustrated similar results as Figure 13a–b after 24 h of immersion time. This can be attributed to the dispersion and the agglomeration of the nanoparticles, as explained above. Hence, after 24 h of immersion, it was observed that the 1G+1${\mathrm{TiO}}_{2}$ coating system revealed the best corrosion performance.

Interestingly, after 30 days of immersion, both Figure 14a,c further revealed that the majority of the capacitive region was present, indicating that the incorporation of 0.5 wt.% and 2 wt.% of ${\mathrm{TiO}}_{2}$ nanoparticles into the polymeric matrix remarkably improved the barrier properties of the coating. However, as illustrated in Figure 14b, the majority area under the Bode plot was observed to be dominated by the resistive region. Moreover, at the highest loading ratio of 3 wt.% ${\mathrm{TiO}}_{2}$ nanoparticles, this coating system exhibited a full capacitive region, thus, confirming that the addition of these inhibitors significantly enhanced the corrosion protection performance of the coating, as illustrated in Figure 14d. Here, it was revealed that the 1G+3${\mathrm{TiO}}_{2}$ coating system had the best corrosion performance after 30 days of immersion. In addition, after 60 days of immersion, Figure 15a,b illustrated the opposite trends whereby higher $f_{b}$ values and the size of the resistive region significantly increased compared to the capacitive region, whereas, Figure 15c,d exhibited a complete capacitive region or having a majority capacitive region over the resistive region, respectively.

However, after 90 days of immersion, Figure 16a,b illustrated a more resistive region compared to the capacitive region. Despite observing such trends, the 1G+0.5${\mathrm{TiO}}_{2}$ and 1G+1${\mathrm{TiO}}_{2}$ the coating system had still demonstrated high ${\left|Z\right|}_{0.01\;\mathrm{Hz}}$ value exceeding $10^{10}$ Ω ${\mathrm{cm}}^{2}$ at low-frequency range throughout the entire period of immersion. Interestingly, only the capacitive region was observed for the coating system containing 2 and 3 wt.% ${\mathrm{TiO}}_{2}$ nanoparticles, as illustrated in Figure 16c,d. In conclusion, due to the non-significant changes in the $f_{b}$ values up to throughout the immersion time, the coating systems containing 2 and 3 wt.% ${\mathrm{TiO}}_{2}$ nanoparticles were observed to be intact and exhibited the optimum results compared to the other coating systems, whereby the coating layer had remarkable stability to successfully prevent the penetration of the corrosive agents.

### 3.7. Surface Morphology

**Figure 17:**
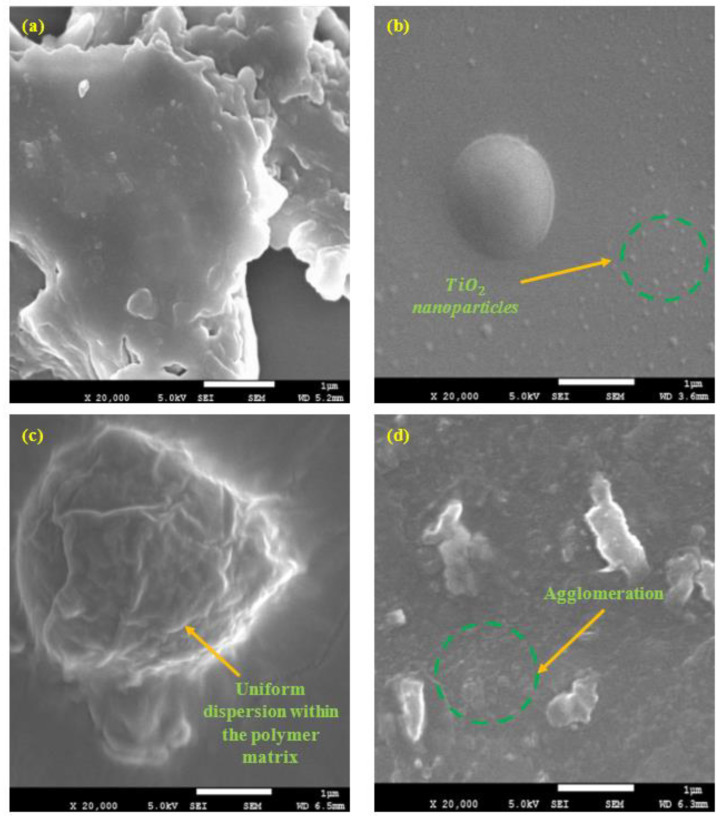

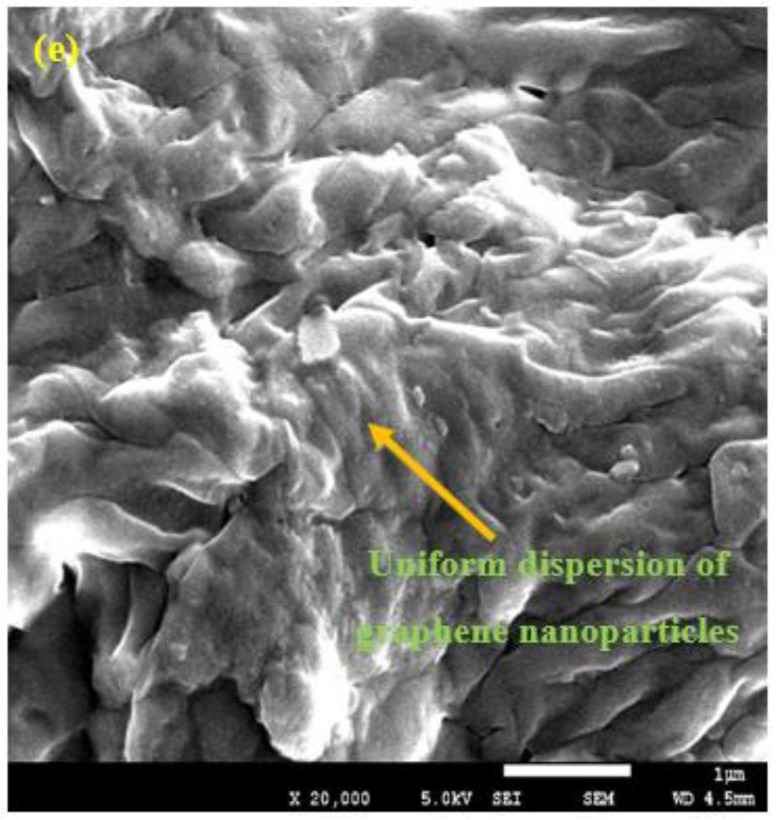
FESEM representations of the fracture surface of the graphene-based polymer nanocomposites coatings, which contained the inclusion of (**a**) 0.5%, (**b**) 1%, (**c**) 2%, (**d**) 3% nanosized ${\mathrm{TiO}}_{2}$ nanoparticles, and (**e**) 1 wt.% graphene coating, respectively.

The surface morphologies of all the graphene/${\mathrm{TiO}}_{2}$ nanocomposite coating films containing different loading ratios of ${\mathrm{TiO}}_{2}$ nanoparticles were investigated by the FESEM, as illustrated in Figure 17a–d. In addition, the nature of the surface, surface failure, distribution of the nanoparticles, dispersion, and phase distribution of the nanoparticles have been indicated in the FESEM micrographs, respectively. Furthermore, without any doubt, it was observed that the surface morphology of all the coated surfaces indicated that the graphene and ${\mathrm{TiO}}_{2}$ nanoparticles homogenously dispersed and distributed within the polymeric matrix. Also, no cracks, defects or phase separation was observed. Hence, this result was in agreement with the previously reported FTIR results, thus, further confirming the efficacy of the curing process [53]. As mentioned before, due to uniformly covering the coating surface, a roughness of the surface was provided by the graphene and ${\mathrm{TiO}}_{2}$, which was accountable for the low surface wettability.

In general, the graphene nanoplatelets consist of an irregular shape, whereas the ${\mathrm{TiO}}_{2}$ nanoparticles have a nanoscopic particle size (<50 µm) [49]. Additionally, specific amounts of air pockets were entrapped on the surface by the interspacing between the protrusions, whereby the inclusion of graphene/${\mathrm{TiO}}_{2}$ within the polymeric matrix introduced protrusions with different interspacing and heights, thus, resulting in the contact point of the liquid on the surface to be minimized. Moreover, it was observed that the porosity factor of the surface had drastically decreased with the addition of these nanofillers and inhibitors. To support these findings, Wu et al. reported that a higher WCA resulted when the solid-liquid interface was at a minimum, thus, resulting in the coating exhibiting hydrophobic or super-hydrophobic characteristics [49].

Furthermore, the coating samples containing 0.5 wt.%, 1 wt.%, and 2 wt.% of ${\mathrm{TiO}}_{2}$ nanoparticles along with the addition of 1 wt.% G was revealed to have a uniform dispersion and distribution of nanoparticles, as illustrated in Figure 17a–c. Among all these hybrid nanocomposite coating systems, the 1 wt.% exhibited the most pronounced dispersion. Here, as indicated (in green) in Figure 17b, the pure ${\mathrm{TiO}}_{2}$ displayed a uniform morphology of tiny spherical nanoparticles. It was observed that the agglomeration of the nanoparticles had occurred as the loading ratio of ${\mathrm{TiO}}_{2}$ increased to 3 wt.%, whereby the presence of relatively larger nanoparticles was observed, as depicted in Figure 17d. Moreover, in accordance with Figure 17e, it was revealed that the inclusion of 1wt.% graphene nanoparticles into the acrylic-epoxy (90A:10E) polymeric matrix had dispersed uniformly, and no obvious agglomeration was found, thus confirming the good adhesion properties of the coating [61]. According to Chen and Zhou et al., the distance between the nanoparticles decreased as the number of nanoparticles increased within the unit area, hence, resulting in the nanoparticles agglomerating at high loading rates [63,64]. In other words, at high loading ratios, this increased the ability of the nanoparticles to attract each other and produce larger agglomerated nanoparticles. In short, these micrographs confirmed the presence of graphene that dispersed well with the ${\mathrm{TiO}}_{2}$ nanoparticles within the polymeric matrix. A larger surface area for the photon absorption was provided by the presence of graphene. The 1G+1${\mathrm{TiO}}_{2}$ coating system revealed the best surface morphology with the good adhesion coating properties.

## 4. Conclusions

At different loading ratios, the ${\mathrm{TiO}}_{2}$ nanoparticles as inhibitors were, respectively, incorporated into the graphene- based hybrid polymeric matrix, and the corrosion protection performance was determined. The FTIR spectra confirmed the cross-linking had occurred between all the organic components and the presence of the ${\mathrm{TiO}}_{2}$ nanoparticles remarkably influenced the structure and properties of the coatings. Furthermore, a two-step trend was exhibited by all the coating systems. Each stage of decomposition was discussed, respectively. Good thermal stability was observed by the coating systems containing 0.5 and 1 wt.% ${\mathrm{TiO}}_{2}$ nanoparticles. Similar results in terms of optical transmittance were observed whereby all the coating systems had absorbed the visible light, hence, resulting in zero percent transmittance. It was also known that due to the scattering of the ${\mathrm{TiO}}_{2}$ nanoparticles within the polymeric matrix, this resulted in a low visible light transmittance. On the other hand, it was also noteworthy to mention that inclusion of ${\mathrm{TiO}}_{2}$ nanoparticles enabled the coating film to gain a high-UV shielding efficiency, which increased with the increment of the loading ratio of ${\mathrm{TiO}}_{2}$ nanoparticles. Moreover, the increment of the loading ratios of ${\mathrm{TiO}}_{2}$ nanoparticles into the polymeric matrix resulted in the WCA of the coating increasing significantly, whereby the coating system containing 3 wt.% ${\mathrm{TiO}}_{2}$ exhibited the highest WCA of 120.85°. In addition, according to the ASTM standard, all the nanocomposite coatings exhibited excellent adhesion behavior of the coatings, receiving 5B and 4B ratings, respectively. The coating systems with 4B ratings were due to the agglomeration of the ${\mathrm{TiO}}_{2}$ nanoparticles at the highest loading ratio (3 wt.%), thereby affecting the bulk properties of the coating film rather than its surface properties. These results were in strong agreement with the FESEM results. It was also revealed that all the coating systems possessed high corrosion resistance, whereby the ${\left|Z\right|}_{0.01\;\mathrm{Hz}}$ value exceeded $10^{10}$ Ω ${\mathrm{cm}}^{2}$ throughout the immersion time. However, among all the coating systems, the 1G+1${\mathrm{TiO}}_{2}$ coating system demonstrated the highest ${\left|Z\right|}_{0.01\;\mathrm{Hz}}$ value over a period of 90 days of immersion. Overall, based on our experimental findings and analysis, we can conclude that the 1G+1${\mathrm{TiO}}_{2}$ coating system (thin in thickness) significantly enhanced the barrier resistance since, in this case, the graphene/${\mathrm{TiO}}_{2}$ coating layer was proven to be more adhesive and intact, thus preventing the development of galvanic cells at the coating/substrate interface.

## Figures and Tables

**Table 1:** Compositions of all the prepared graphene/${\mathrm{TiO}}_{2}$ coating systems along with their corresponding nomenclature.

Sample Code	Acrylic Resin (wt.%)	Epoxy Resin (wt.%)	Graphene Nanoparticles (wt.%)	${\mathrm{TiO}}_{2}$ Nanoparticles (wt.%)	PDMS (wt.%)	Dry Coating Film Thickness, µm (Avg.)
1G+0.5${\mathrm{TiO}}_{2}$	90	10	1	0.5	1	80
1G+1${\mathrm{TiO}}_{2}$	90	10	1	1	1	88
1G+2${\mathrm{TiO}}_{2}$	90	10	1	2	1	99
1G+3${\mathrm{TiO}}_{2}$	90	10	1	3	1	124

**Table 2:** The TGA parameters—initial degradation temperature (IDT), temperature of 50% weight loss rate and the char residue yield at 700 °C for all the coating systems.

Coating System	Initial Degradation Temperature, IDT (°C)	$\mathrm{Temperature}\;\mathrm{of}\;50\%\;\mathrm{Weight}\;\mathrm{Loss},\;T_{50wt\%}$	Residue Yield (%) at 700 °C
90A:10E	293.23	399.82	0.041
1G	280.41	388.81	2.177
1G+0.5TiO_2_	282.76	405.16	3.064
1G+1.0TiO_2_	280.43	406.71	2.748
1G+2.0TiO_2_	290.31	402.84	3.345
1G+3.0TiO_2_	288.57	403.62	3.942

**Table 3:** The recorded ${\left|Z\right|}_{0.01\;\mathrm{Hz}}$ values for all the graphene/${\mathrm{TiO}}_{2}$ coating systems from the first up to 90 days of immersion.

Coating System	${\left|Z\right|}_{0.01\;\mathrm{Hz}}$ $\;\mathrm{Values}\;(Ω\;cm^{2})$			
	1st Day	30th Day	60th Day	90th Day
1G+0.5TiO_2_	1.03 × $10^{10}$	1.11 × $10^{10}$	9.10 × $10^{9}$	1.20 × $10^{10}$
1G+1TiO_2_	1.44 × $10^{10}$	1.56 × $10^{10}$	1.80 × $10^{10}$	2.13 × $10^{10}$
1G+2TiO_2_	1.12 × $10^{10}$	1.18 × $10^{10}$	1.52 × $10^{10}$	1.69 × $10^{10}$
1G+3TiO_2_	1.11 × $10^{10}$	1.16 × $10^{10}$	1.33 × $10^{10}$	1.40 × $10^{10}$

## Data Availability

Not applicable.

## Supplementary Materials

The following supporting information can be downloaded at: https://www.mdpi.com/article/10.3390/polym15112428/s1; Table S1: Material specifications of acrylic polyol resin (Product Name: Desmophen A 870 BA); Table S2: Material specifications of NCO curing agent (Product Name: Desmodur N 75 MPA/X); Table S3: Material specifications of epoxy resin (Product Name: NPSN-901x75).
