# Supplementary material for: Anticorrosion, Thermal Degradation, and Hydrophobic Performances of Graphene/TiO2 Nanocomposite Coatings
Source: Polymers (Basel). 2023 May 23;15(11):2428. doi: 10.3390/polym15112428 (PMC10255546; doi:10.3390/polym15112428)
Supplement: Supplementary file 1 [file polymers-15-02428-s001.zip › polymers-2294940-supplementary.pdf]

polymers

MDPI

Supplementary Information

Anticorrosion, Thermal Degradation, and Hydrophobic Performances of Graphene/TiO_2 Nanocomposite Coatings

Sachin Sharma Ashok Kumar [1], Nujud Badawi Mohammed[1], Osamah Alduhaish [2], Kasi Ramesh [1,*], Subramaniam Ramesh [1], Mujeeb Khan [2], Baji Shaik [3] and Syed. F. Adil [2,*]

[1] Centre for Ionics University of Malaya, Department of Physics, Faculty of Science, Universiti Malaya, Kuala Lumpur 50603, Malaysia; sachinrahulsharma@gmail.com (S.S.A.K.); lyde467@hotmail.com (N.B.M.); ramesh@um.edu.my (S.R.)
[2] Chemistry Department, College of Science, King Saud University, P.O. 2455, Riyadh 11451, Saudi Arabia; oalduhaish@ksu.edu.sa (O.A.); kmujeeb@ksu.edu.sa (M.K.)
[3] School of Chemical Engineering, Yeungnam University, Gyeongsan 38541, Republic of Korea; shaikbaji@yu.ac.kr
* Correspondence: Author: rameshkasi@um.edu.my (K.R.); sfadil@ksu.edu.sa (S.F.A.)

Table S1: Material specifications of acrylic polyol resin (Product Name: Desmophen A 870 BA).

Material Specifications	Value	Unit of measurements
Viscosity at 23 ºC	3500 ± 700	mPA.s
Density at 20 ºC	1.09	g/mL
Hydroxyl content	2.95 ± 0.15	%
Butyl acetate - Solvent		-
Non-volatiles (Solid content wt.%)	70 ± 1	%

polymers MDPI

Table S2: Material specifications of NCO curing agent (Product Name: Desmodur N 75 MPA/X).

Material Specifications	Value	Unit of measurements
Viscosity at 23 ºC	250 ± 75	mPA.s
Density at 20 ºC	1.07	g/cm^3
NCO content	16.5 ± 0.3	%
Solvent - 1-methoxypropylacetate-2/xylene 1:1		-
Non-volatiles (Solid content wt.%)	75 ± 1	%

Table S3: Material specifications of epoxy resin (Product Name: NPSN-901x75).

Material Specifications	Value	Unit of measurements
Viscosity at 25 ºC	8000-15000	mPA.s
Density at 25 ºC	1.1	g/cm^3
Epoxy equivalent weight	450-500	g/eq
Solvent - Xylene		-
Non-volatiles (Solid content wt.%)	74-76	%
